# Cell division cycle associated 8: A novel diagnostic and prognostic biomarker for hepatocellular carcinoma

**DOI:** 10.1111/jcmm.17032

**Published:** 2021-11-05

**Authors:** Xiao‐Han Cui, Qiu‐Ju Peng, Ren‐Zhi Li, Xia‐Jie Lyu, Chun‐Fu Zhu, Xi‐Hu Qin

**Affiliations:** ^1^ Department of General Surgery The Affiliated Changzhou No. 2 People’s Hospital of Nanjing Medical University Changzhou Jiangsu China; ^2^ Nanjing Medical University Nanjing Jiangsu China; ^3^ Department of Pediatrics The Affiliated Changzhou No. 2 People’s Hospital of Nanjing Medical University Changzhou Jiangsu China; ^4^ Weifang Medical University Weifang Shandong China

**Keywords:** biomarker, Cell Division Cycle Associated 8 (CDCA8), diagnosis, E2F1, hepatocellular carcinoma (HCC), prognosis

## Abstract

The cell division cycle associated 8 (CDCA8) is a crucial component of the chromosome passenger complex (CPC). It has been implicated in the regulation of cell dynamic localization during mitosis. However, its role in hepatocellular carcinoma (HCC) is not clearly known. In this study, data of 374 patients with HCC were retrieved from the Cancer Genome Atlas (TCGA) database. Pan analysis of Gene Expression Profiling Interactive Analysis (GEPIA) database was performed to profile the mRNA expression of CDCA8 in HCC. Then, the Kaplan‐Meier plotter database was analysed to determine the prognostic value of CDCA8 in HCC. In addition, samples of tumour and adjacent normal tissues were collected from 88 HCC patients to perform immunohistochemistry (IHC), reverse transcription‐quantitative polymerase chain reaction (qRT‐PCR) and Western blotting. The results obtained from bioinformatic analyses were validated through CCK‐8 assay, EdU assay, colony formation assay, cell cycle assays and Western blotting experiments. Analysis of the Kaplan‐Meier plotter database showed that high expression of CDCA8 may lead to poor overall survival (OS, p = 4.06e‐05) in patients with HCC. For the 88 patients with HCC, we found that stages and grades appeared to be strongly linked with CDCA8 expression. Furthermore, the high expression of CDCA8 was found to be correlated with poor OS (*p *= 0.0054) and progression‐free survival (PFS, *p *= 0.0009). In vitro experiments revealed that inhibition of CDCA8 slowed cell proliferation and blocked the cell cycle at the G0/G1 phase. In vivo experiments demonstrated that inhibition of CDCA8 inhibited tumour growth. Finally, blockade of CDCA8 reduced the expression levels of cyclin A2, cyclin D1, CDK4, CDK6, Ki67 and PCNA. And, there is an interaction between CDCA8 and E2F1. In conclusion, this research demonstrates that CDCA8 may serve as a biomarker for early diagnosis and prognosis prediction of HCC patients. In addition, CDCA8 could be an effective therapeutic target in HCC.

## 1 INTRODUCTION

With a rapidly increasing global incidence and postoperative recurrence rate, liver cancer is the fifth most common type of cancer, accounting for the second‐highest number of cancer‐related deaths.^1^, ^2^ Hepatocellular carcinoma (HCC) is the most predominant histological type of liver cancer, accounting for about 80% of all liver cancers.^1^ In 2021, HCC‐associated morbidities and mortalities were 42230 and 30230, respectively, in the United States.^3^ However, only 30% of patients were diagnosed at an early stage and received appropriate treatments, including resection, chemotherapy or liver transplantation.^3^ However, clinical diagnosis is often delayed, with advanced HCC accounting for most of the diagnosed cases.^4^ Advances in HCC prevention and treatment have not improved the clinical outcomes for HCC, with a 5‐year overall survival rate of only 15%.^5^ Therefore, there is a need to identify sensitive biomarkers to inform the development of novel therapeutic strategies.

The occurrence and progression of HCC are associated with various factors, especially the accumulation of genetic variations. The cell division cycle‐related protein family, including cell division cycle associated 1–8 (CDCA1–8), is an essential regulator of cell proliferation.^6^ During the cell cycle, chromosomal passenger complex (CPC) plays important roles in right chromosome segregation and cytokinesis. The CPC is made up of enzymatic core Aurora‐B kinase, scaffold protein inner centromere protein, CDCA8 and two other non‐enzymatic surviving subunits.^7^ Therefore, CDCA8 may be an important component in mitosis regulation.^8^, ^9^ Elevated CDCA8 levels are associated with poor prognostic outcomes for breast, gastric, kidney and bladder cancers.^10^, ^11^, ^12^ Moreover, overexpressed CDCA8 is critical for the growth of embryonic stem cells and breast cancer.^13^ CDCA8 induces tamoxifen resistance in breast cancer cells and promotes their proliferation.^14^ Moreover, CDCA8 affects the proliferation of breast cancer cell lines by directly mediating E2.^14^ Currently, the role of CDCA8 in HCC has not been elucidated. We aimed at determining the significance of CDCA8 as a prognostic and therapeutic target for HCC.

The Cancer Genome Atlas (TCGA) is the largest cancer database, with more than 20,000 primary cancer samples and paired paracancer samples for 32 cancer types. Therefore, the genetic data for HCC can be obtained from this database and evaluated through bioinformatics methods. To assess the relationship between CDCA8 and HCC progression, raw data were processed by multi‐dimensional analysis using the R software. Finally, findings from bioinformatics analyses were validated by in vivo and in vitro experiments.

## 2 METHODS

### 2.1 Data acquisition and identification of differentially expression mRNAs

Transcriptome data for HCC, including 50 normal tissues and 374 HCC tissues, were downloaded from the TCGA database. Clinical information, including age, gender, stage, pathological information and OS, was obtained from TCGA‐LIHC. Transcriptome data were subjected to differential analysis using the edgeR package. Significant criteria were |log 2 (fold change [FC])| > 1 and *p *< 0.05.

### 2.2 Screening for independent prognostic genes and core node

Protein‐protein interaction (PPI) network analysis is essential for identification of molecules that mediate vital cellular processes in HCC progression. We established the PPI networks of the top 9 pivotal genes with high connectivities. The PPI data were obtained by querying independent prognostic gene queries from public databases. The Cytoscape plugin was used for network visualization, analysis and publication.

### 2.3 GEPIA Database Analysis

Transcriptional profiles of CDCA8 in multiple cancers were obtained from the GEPIA database (http://gepia.cancer‐pku.cn/index.html). TCGA tumour versus TCGA normal +The Genotype‐Tissue Expression (GTEx) normal data set was used to establish the expression box plots. The log2FC cut‐off was set to 1, while the p‐value cut‐off was set to 0.01. Genes with higher |log2FC| values and p‐values below a preset threshold were considered to be differentially expressed genes (DEGs).

### 2.4 The Human Protein Atlas (HPA)

The HPA provides information on tissue and cellular distributions of 26,000 human proteins. Using this database, expression levels of each protein in 64 cell lines, 48 normal human tissues and 20 tumour tissues were evaluated using immunoassay techniques. To establish the expression and location of CDCA8 in normal liver and HCC tissues, CDCA8‐related information was searched in HPA.

### 2.5 Gene set variation analysis

Gene set variation analysis (GSVA) analysis was performed by an unmonitored gene enrichment method to calculate changes in pathway activities between samples in an unsupervised manner using the R software. High‐ and low expression groups were defined as experimental and control groups, respectively, and the Hallmark gene set was used for comparisons. The threshold was set at DR < 0.25 and *p *< 0.05. The criteria for gene sequencing were Signal2Noise.

### 2.6 Cell type identification by estimating relative subsets of RNA transcripts

Cell type identification by estimating relative subsets of RNA transcripts (CIBERSORT) allows for the analysis of immune cell characteristics by unscrambling gene expression microarray data. We evaluated the proportion of immune cells in HCC tissue samples with high‐ and low expression levels of CDCA8.

### 2.7 Sample Collection

Both HCC and normal tissues were obtained from HCC patients who had been subjected to surgical resection at the Second People's Hospital of Changzhou between 2014 and 2016. Pathologically confirmed HCC patients were included in this study, while patients with other types of malignancies were excluded. Finally, 88 HCC patients were included in the cohort. Samples for RNA and protein extraction were freshly frozen in liquid nitrogen and stored at −80°C. HCC and their paired non‐tumour samples for immunohistochemical analysis were formalin‐fixed. The Research Ethics Committee of the Second People's Hospital of Changzhou approved this study, and all patients were required to sign informed consents.

### 2.8 Cell Culture and Treatment

Human HCC cell lines (Huh7, Hep‐3B, Hep‐G2, MHCC‐LM3, MHCC‐97L and MHCC‐97H) were purchased from the Chinese Academy of Sciences (Shanghai, China). They were cultured in DMEM media supplemented with 10% foetal bovine serum (FBS) (Gibco, Grand Island, NY, USA) and incubated at 37°C in a 5% CO_2_ atmosphere.

### 2.9 Reverse transcription‐quantitative polymerase chain reaction (RT‐QPCR) and Plasmids

Total RNA was extracted from liver tissues and cell lines using the E.Z.N.A. Total RNA Kit I (Omega Bio‐tek, GA, USA), according to the manufacturer's instructions. The extracted RNA was reverse transcribed into complementary DNA (cDNA) using HisScript Ⅱ (Vazyme, Shanghai, China). qRT‐PCR was performed using SYBR Green Ⅰ (Vazyme, Shanghai, China) on an ABI 7900 system. The CDCA8 primers used in this study are shown in Table S1. For CDCA8 knockdown, the target shRNA sequence (Table S1) was subcloned into the pLVX‐shRNA Lentivector. E2F1 and CDCA8 CDS sequences were loaded into the PCDH plasmid to induce the overexpression of E2F1 and CDCA8 in HCC cells. To generate a recombinant lentivirus, recombinant lentiviral plasmids were co‐transfected with pMD2G and pSPAX2 into HEK293T cells. For lentiviral infection, Huh7 and Hep‐3B were seeded in 6‐well plates and incubated to 70–80% confluence. Then, they were transfected with the lentivirus. Two days later, puromycin was added for screening. Stable cells were cultured in DMEM medium containing 10% FBS. Knockdown efficiencies of CDCA8 were assessed by RT‐PCR and Western blot.

### 2.10 Western Blotting

The RIPA lysis buffer (Beyotime Biotechnology, Shanghai, China) was used to lyse the HCC cell lines and liver tissues. Then, proteins were collected and quantified using the bicinchoninic acid (BCA) kit (Beyotime Biotechnology, Shanghai, China). Proteins were isolated by sodium dodecyl sulphate (SDS)‐PAGE on 10% gels and transferred to polyvinylidene fluoride (PVDF) membranes (Sigma‐Aldrich, St. Louis, MO, USA). After overnight incubation with various primary antibodies, including anti‐CDCA8 (1:1000, 12465–1‐AP, proteintech), Cyclin A2 (1:2000, 66391–1‐lg, proteintech), Cyclin D1(1:5000, 60186–1‐lg, proteintech), CDK4 (1:1000, 11026–1‐AP, proteintech), CDK6 (1:1000, 14052–1‐AP, proteintech), Ki67 (1:1000, ab16667, Abcam), PCNA (1:1000, 10205–2‐AP, proteintech), E2F1 (1:1000,66515–1‐Ig, proteintech) and anti‐GAPDH antibody (1:2500, ab9485, Abcam) at 4℃, membranes were washed thrice for 5 min each time, using TBST (in 0.1% Tween20). Then, they were incubated in the presence of a secondary rabbit antibody (1:2000, A0208, Beyotime Biotechnology) for 1 h and washed thrice using TBST for 5 min each time. Signals were detected using the chemiluminescence system and analysed by the ImageJ Lab software.

### 2.11 Immunohistochemistry (IHC)

Paraffinized tissue sections from 88 HCC patients were used for IHC staining. After routine steps, the tissues were stained with a CDCA8 (1:200, Proteintech, Chicago, IL, USA) primary antibody. Intensities of positive staining were defined as 0, 1+, 2+ and 3+, indicating no, weak, moderate and strong staining, respectively. Staining distribution was expressed as the percentage of positive tumour cells (0% to 100%). The two variables were multiplied to obtain the final CDCA8 expression score. Based on CDCA8 expression scores, samples were further divided into low (0, 1+) and high (2+, 3+) expression groups.

Cell Counting Kit‐8 assay As instructed by the manufacturer, the Cell Counting Kit‐8 (CCK‐8) assay (MedChemExpress) was used to evaluate cell absorbance and compare cellular proliferations. Huh7 and Hep‐3B cells were seeded in 96‐well plates (5 × 10^3^ cells/well) and incubated at 37°C in a 5% CO_2_ environment. Then, 100 µl of DMEM medium containing 10% CCK‐8 solution was simultaneously added to each well. Optical densities (OD) were measured at 450 nm using a microplate reader (Thermo Fisher Scientific, Inc.) at the same time point from 0 to 4 days.

### 2.12 Colony formation assay

Huh7 and Hep‐3B cells were seeded in 6‐well plates (1 × 10^3^ cells/well) and incubated for 14 days. Cells were fixed for 15 min in 4% paraformaldehyde and stained with 1% crystal violet for 1 min after which colonies were counted.

### 2.13 The 5‐ethynyl‐2′‐deoxyuridine (EdU) assay

The EdU assay was performed by aCell‐Light EdU Apollo567 In Vitro Kit (RiboBio, Guangzhou, China), according to the manufacturer's instructions.

### 2.14 Cell cycle analysis

Cell cycle analysis was performed using a Cell Cycle Staining Kit (MultiSciences, Hangzhou, China), as instructed by the manufacturer. Cells were washed using PBS, after which 1 ml of DNA staining solution and 10 μl of permeate were added to the cell suspension and vortexed to mix. Finally, cells were stained in the dark at 4°C for 30 min and analysed by flow cytometry.

### 2.15 Apoptotic analysis

The apoptosis assay was performed using the Annexin V‐FITC/PI apoptosis detection kit (Beyotime, Shanghai, China), according to the manufacturer's protocol. First, cells were harvested and washed twice using cold PBS. Then, they were suspended in 400 μl of binding buffer, 5 μl of Annexin V antibody conjugated with fluorescein isothiocyanate (FITC) and 5 μl of PI solution. Samples were stained for 15 min at room temperature in the dark, after which the apoptotic cell ratio was determined by flow cytometry (Beckman Coulter, FL, USA).

### 2.16 Animal breeding and treatments

For in vivo tumorigenicity assays, 3 × 10^6^ cells, including Huh7and MHCC‐LM3 with the recombinant lentivirus were harvested and resuspended in 100 μl of PBS and subcutaneously injected into 4‐ to 6‐week‐old male BALB/c nude mice. Tumour volumes were measured as (tumour width^2^ × tumour length)/2 and recorded every 4 days. Mice were sacrificed at 36 days post‐inoculation. Finally, subcutaneous tumours were excised and further analysed. All male nude mice (BALB/c) were obtained from GemPharmatech Co. Ltd., Nanjing, China, and housed in a 12 h light/dark cycle at 23–26°C.

### 2.17 Statistical analysis

Data analysis was performed using the SPSS 25.0 software and GraphPad Prism 5.0. Chi‐square or Fisher's exact tests were used to assess the relationships between protein expression levels and clinic‐pathological information. Kaplan‐Meier curves and log‐rank tests were used to assess progression‐free survival (PFS) and overall survival (OS) outcomes. Student's t tests were used to establish differences between the two groups, and *p *< 0.05 was the threshold for statistical significance.

## 3 RESULTS

### 3.1 Screening for DEGs and selection of core genes

**FIGURE 1:**
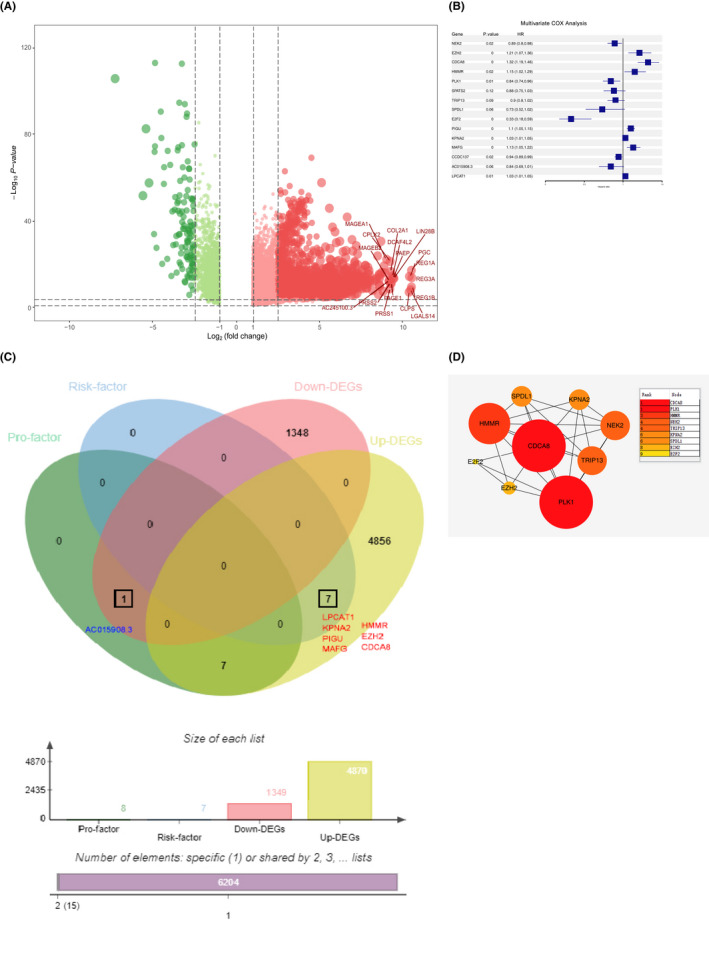
Screening results for differentially expressed genes. (A) Volcano maps of gene expression in HCC and normal tissues. Data points in red and green represent upregulated and downregulated genes, respectively. (B) Screening for independent prognostic genes using multifactorial cox analysis. (C) In HCC, seven upregulated proteins were associated with risk factor. (D) CDCA8 was the core gene in the PPI network

To screen for DEGs, TCGA‐LIHC transcriptome data for 50 cases of normal tissue and 374 cases of HCC tissues were downloaded. DEGs, including upregulated (red) and downregulated (green) genes are shown by volcano plots (Figure 1A). Multivariate Cox analysis was used to screen independent prognostic genes (Figure 1B). The upregulated DEGs and risk factor‐related genes were overlapped after which LPCAT1, HMMR, EZH2, CDCA8, KPNA2, PIGU and MAFG were screened (Figure 1C). Finally, protein interaction (PPI) network analysis of independent prognostic genes revealed that CDCA8 was the most significant core gene in the PPI network (Figure 1D). Therefore, we performed comprehensive bioinformatic analyses and experimental validation for CDCA8.

### 3.2 CDCA8 is Upregulated in HCC and Correlates with HCC Prognosis

**FIGURE 2:**
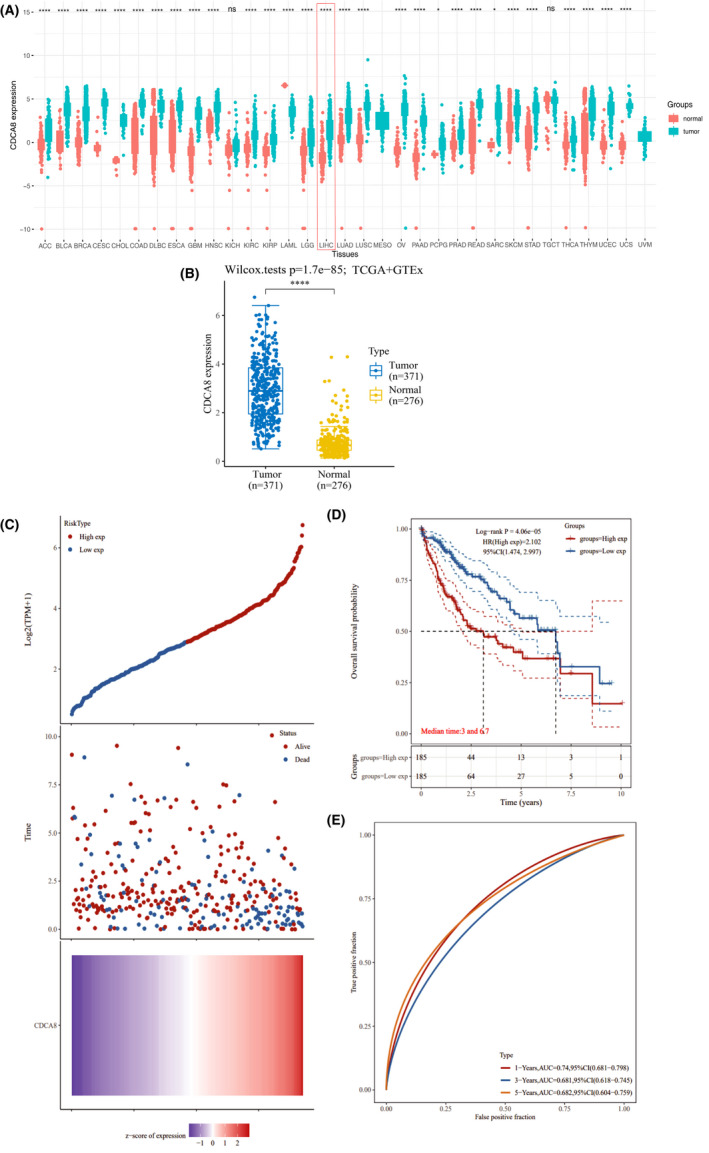
CDCA8 is upregulated in HCC and is associated with poor prognosis. (A) In the TCGA database, CDCA8 was found to be differentially expressed in various tumours and significantly upregulated in HCC. (B) In TCGA and GEO databases, expression levels of CDCA8 were significantly upregulated in HCC. (C) The risk score for each HCC patient increased from blue to red, survival time for each HCC patient and the CDCA8‐related signature. (D) In HCC patients, upregulated CDCA8 were associated with poor prognosis. (E) Time‐dependent ROC curves for OS of the CDCA8‐related signature score at 1‐, 3‐ and 5‐year period. ^*^ *p *< 0.05, ^**^ *p *< 0.01 and ^***^ *p *< 0.001. *p *< 0.05 was considered statistically significant

Using the GEPIA database, we established that CDCA8 was upregulated in various malignancies, especially HCC (Figure 2A). Based on TCGA and GTEx databases, CDCA8 was found to be significantly upregulated in HCC tissues (Figure 2B). To visualize the expression levels of CDCA8 in normal liver tissues and HCC, we downloaded three typical IHC samples of CDCA8 from normal liver tissues and liver cancer tissues in HPA (Supplement Material Figure S1).

In the TCGA database, expression levels of CDCA8 in patients were allocated into high‐ and low expression groups according to the median value of CDCA8 expression levels in each HCC patient (Figure 2C). Then, we plotted the Kaplan‐Meier curves using the TCGA data and found that elevated CDCA8 levels were associated with poor OS outcomes (Figure 2D). Finally, ROC curves were used to establish the reliability of the prognostic model (1‐year, AUC = 0.74, 3‐year, AUC = 0.681, 5‐year, AUC = 0.682) (Figure 2E).

### 3.3 Validation of the Association between Aberrantly Expressed CDCA8 and Prognosis of Clinical HCC Cohorts

**FIGURE 3:**
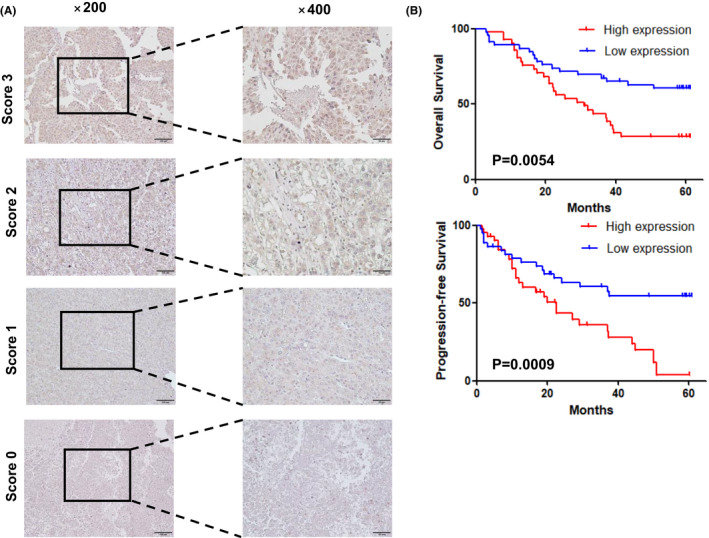
Representative images of HCC tissues, and the relationship between high and low expressions of CDCA8 with OS and PFS. (A) Scores indicate CDCA8 expression levels in representative HCC tissues. The fraction was calculated from the intensity and percentage of stained cells as described in the methods. (B) The prognostic significance of CDCA8 in HCC patients was assessed by Kaplan‐Meier analysis. Patients with low CDCA8 expressions (score 0–1) had better OS outcomes and lower probabilities of recurrence compared to those with high CDCA8 expressions (score 2–3)

**FIGURE 4:**
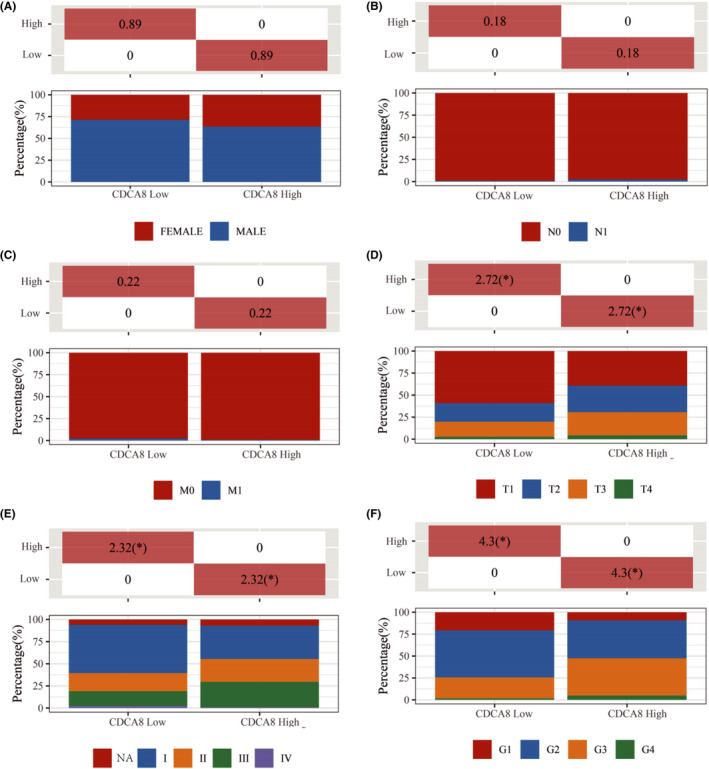
The association between CDCA8 expression levels and clinic‐pathologic characteristics in the TCGA data set. (A) Upregulated CDCA8 levels were not associated with gender. (B) Upregulated CDCA8 levels were not associated with lymph node metastasis. (C) Upregulated CDCA8 levels were not associated with distant metastasis. (D) Upregulated CDCA8 levels were associated with poor T stage. (E) Upregulated CDCA8 levels were associated with poor pathological stage. (F) Upregulated CDCA8 levels were associated with poor histopathologic grade. ^*^ *p *< 0.05, ^**^ *p *< 0.01 and ^***^ *p *< 0.001. *p *< 0.05 was considered statistically significant

To validate the results of Kaplan‐Meier curves that had been plotted using the TCGA database, 88 HCC samples were subjected to IHC staining and evaluated by the E‐score criteria. Based on the staining intensity of the CDCA8 antibody and staining range, we calculated the E‐scores of each sample (Figure 3A). The scores represented protein abundances of CDCA8 in the liver tissue. Based on expression levels of CDCA8, clinical and pathological information of each sample were obtained. Then, based on the clinical cohort, we visualized the OS and PFS outcomes of patients and found that elevated CDCA8 levels were associated with poor OS (*p*=0.0054) and PFS (*p *= 0.0009) outcomes (Figure 3B). To verify the validity of the plotted Kaplan‐Meier curves, we performed statistical analyses of baseline information of the TCGA‐LIHC data set and data for 88 samples, respectively. Using the TCGA‐LIHC data set, correlations between CDCA8 levels and gender, TNM stage, pathological stage and grade were assessed (Figure 4A‐F). There were significant correlations between CDCA8 levels and T stage, pathological stage as well as grade (Figure 4D‐F). Moreover, CDCA8 levels were found to be independent of age (*p *= 0.682), gender (*p *= 0.43), α‐fetoprotein (AFP) serum levels (*p *= 0.516), liver fibrosis (*p *= 0.29) and hepatitis B (*p *= 0.317). Upregulated CDCA8 levels were significantly positively correlated with poor TNM stage (*p *= 0.028) and tumour size (*p *= 0.005) (Table 1). Therefore, we postulated that CDCA8 is likely to function as an oncogene in HCC progression.

**TABLE1. 1:** Correlations between the expression of CDCA8 and clinicopathological features in 88 HCC patients

Characteristics	Case	CDCA8 expression		*p*
		Low	High	
All cases	88	46	42	
Age(years)				
<50	27	12	30	0.682
≥50	61	15	31	
Gender				
Male	36	23	19	0.43
Female	52	29	17	
pTNM stage				
Ⅰ&Ⅱ	29	20	9	0.028
Ⅲ	59	26	33	
Tumour size(cm)				
<5	32	23	9	0.005
≥5	56	23	33	
α‐fetoprotein(ng/ml)				
<20	45	22	23	0.516
≥20	43	24	19	
Liver cirrhosis				
Yes	45	26	19	0.29
No	43	20	23	
Hepatitis Bs antigen				
Yes	53	30	23	0.317
No	35	16	19	

* *p *< .05.

### 3.4 Underlying Biological Pathways in which CDCA8 is enriched

**FIGURE 5:**
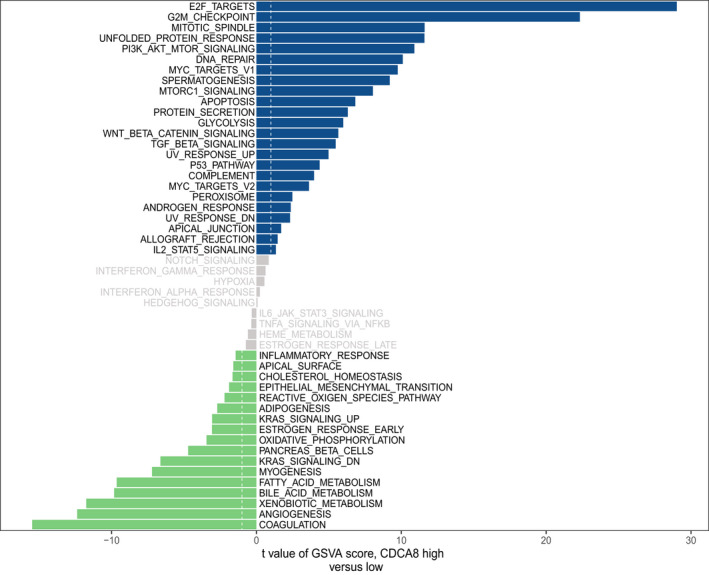
Up‐ and downregulation of CDCA8 was associated with activations of multiple pathways, respectively. Upregulation of CDCA8 activated E2F targets, G2 M checkpoint, mitotic spindle, unfolded protein response, PI3K AKT mTOR signalling and DNA repair among others. Downregulation of CDCA8 activated coagulation, angiogenesis, bile acid metabolism, fatty acid metabolism, myogenesis, kras signalling DN and pancreas beta cells among others

The CDCA family is involved in cell cycle progression and cell proliferation. Therefore, through GSVA, we assessed the downstream pathways for CDCA8. Upregulated CDCA8 was shown to activate the E2F targets, G2 M checkpoint, mitotic spindle, unfolded protein response, PI3K AKT mTOR signalling and DNA repair among others (Figure 5). Downregulation of CDCA8 was associated with activated coagulation, angiogenesis, bile acid metabolism, fatty acid metabolism, myogenesis, KRAS signalling DN and pancreatic beta cells among others (Figure 5). As a potential oncogene, the pathways activated by CDCA8 upregulation might be involved in its effects on HCC progression.

### 3.5 Correlation between CDCA8 and immune infiltrating cells

**FIGURE 6:**
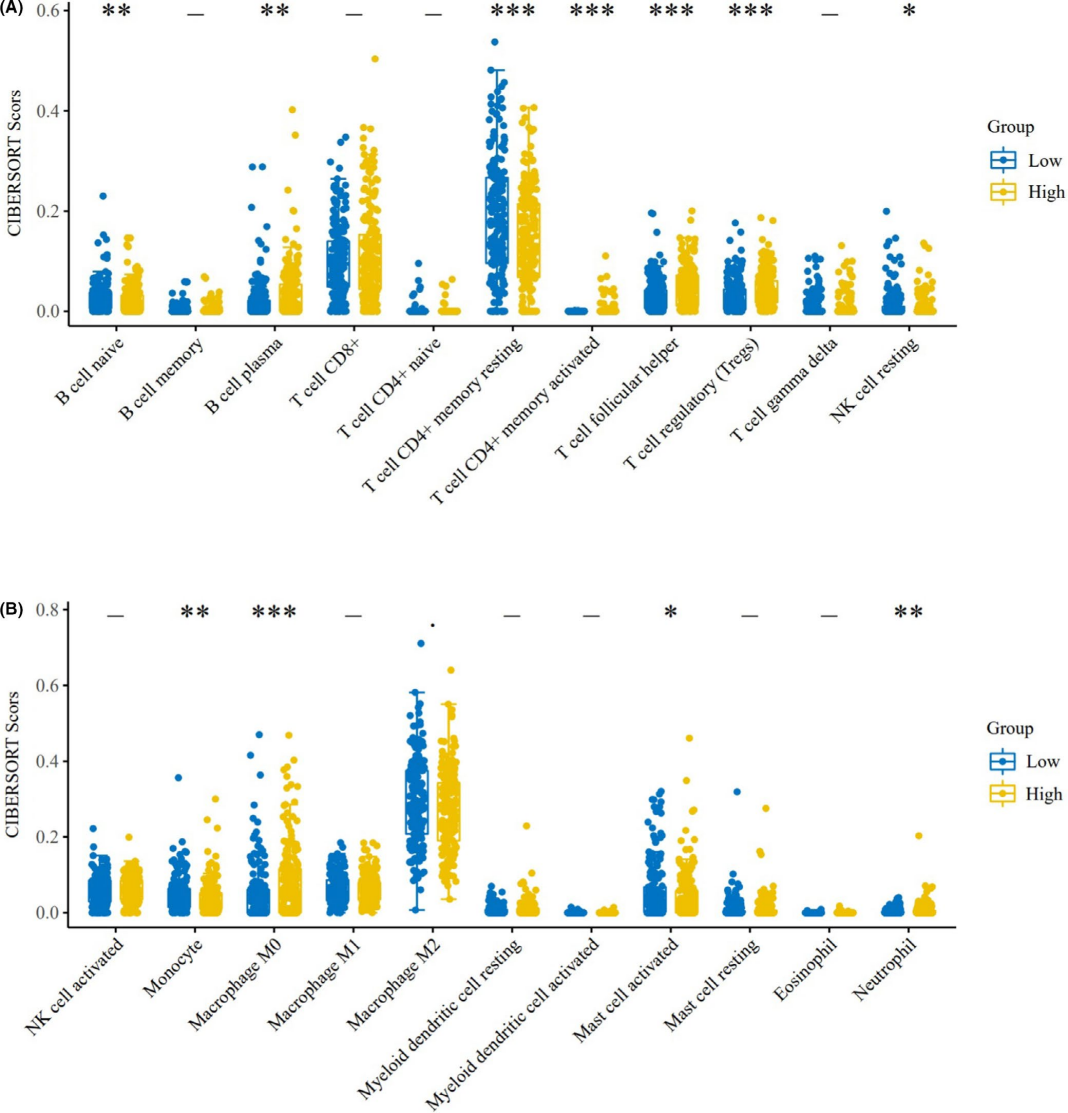
Upregulated or downregulated CDCA8 correlated with the CIBERSORT Score of multiple immune infiltrating cells. (A) Differential expression of CDCA8 correlated with infiltrations of naive B cells, plasma B cells, resting memory CD4+ T cells, activated memory CD4+ T cells, follicular helper T cells, regulatory T cells (Tregs) and resting NK cells. (B) Differential expressions of CDCA8 correlated with monocyte, macrophage M0, activated mast cell and neutrophil infiltrations. ^*^ *p *< 0.05, ^**^ *p *< 0.01 and ^***^ *p *< 0.001. *p *< 0.05 was considered statistically significant

Figure 5 shows that CDCA8 is associated with E2F targets and IL2 STAT5 signalling. Therefore, CDCA8 may be involved in immune‐related processes in HCC. Correlation analyses of CDCA8 levels and infiltrating immune cells were performed to investigate the significance of CDCA8 in immunity. Differential expressions of CDCA8 were correlated with naive B cell, plasma B cell, resting memory T cell CD4+, activated memory T cell CD4+, helper T cell follicular, regulatory T cell (Tregs), resting NK cell, Monocyte, Macrophage M0, activated mast cell and neutrophil levels (Figure 6A, B).

### 3.6 Suppressed CDCA8 affected HCC cell proliferation and cell cycle progression

**FIGURE 7:**
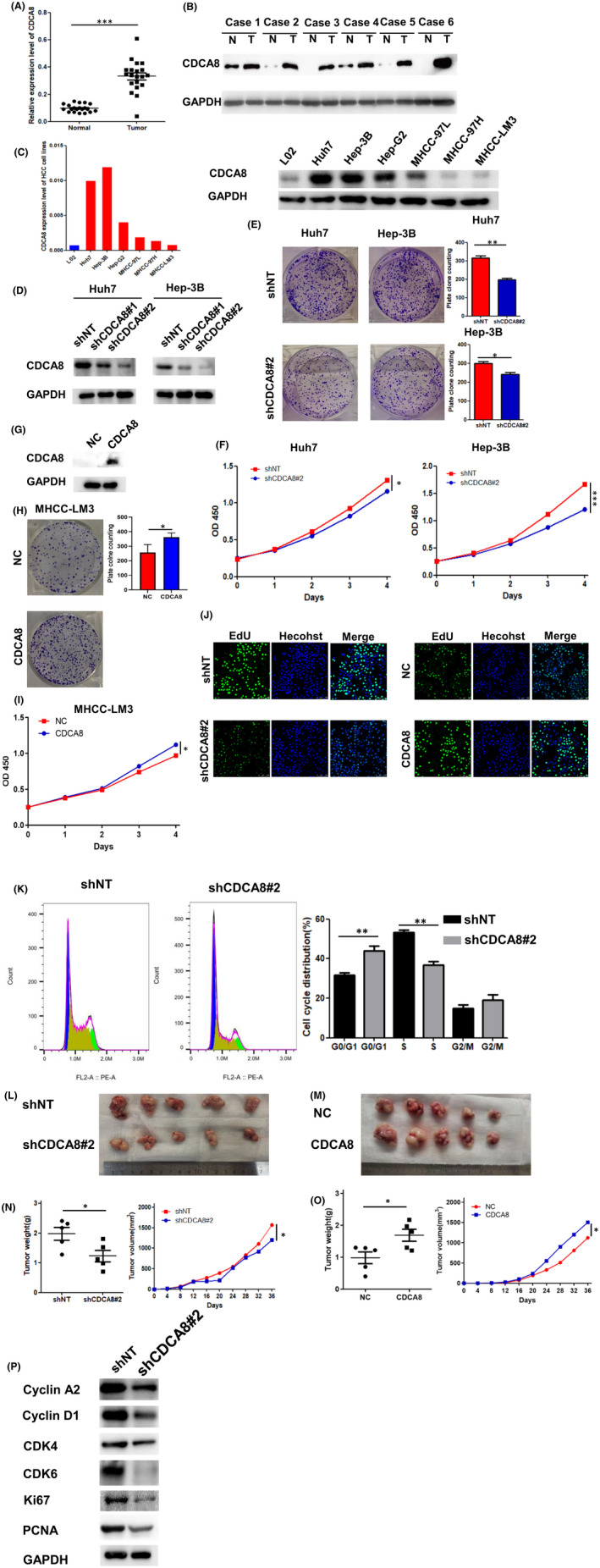
CDCA8 expression level was elevated in HCC. Knockdown of CDCA8 inhibited the proliferation of HCC cells. (A‐B) The expression level of CDCA8 was upregulated in HCC. (C) CDAC8 was expressed in a variety of HCC cell lines. (D) Stable knockdown of CDCA8 in Huh7 and Hep‐3B cell lines. (E) Results of the cloning assay showed that knockdown of CDCA8 inhibited the proliferation of Huh7 and Hep‐3B cells. (F) Knockdown of CDCA8 inhibited the proliferation of Huh7 and Hep‐3B cells as determined by CCK‐8 assay. (n = 3) (G) Stable overexpression of CDCA8 in MHCC‐LM3 cell lines. (H) Overexpression of CDCA8 increased the proliferation of MHCC‐LM3 as shown by the plate cloning assay. (n = 3) (I) Results of the CCK‐8 assay revealed that overexpression of CDCA8 increased the proliferation of MHCC‐LM3 cell lines. (n = 3) (J) Knockdown of CDCA8 decreased the proliferation of Huh7 cells, and overexpression of CDCA8 increased the proliferation of MHCC‐LM3 cells as determined by EdU assay. (n = 3) (K) Flow assay indicated that knockdown of CDCA8 inhibited the cell cycle at G0/G1 in Huh7 cells. (L, N) Knockdown of CDCA8 decreased the tumour volume and weight of xenografts in vivo. Images showing the size of tumours in mice. (n = 5) (M, O) Overexpression of CDCA8 increased tumour volume and weight of xenografts in vivo. Images showing the size of tumours in mice. (n = 5) (P) Knockdown of CDCA8 altered expression of cell cycle‐related proteins. Data are presented as the mean ± SD of at least three independent experiments. ^*^ *p *< 0.05, ^**^ *p *< 0.01 and ^***^ *p *< 0.001. *p *< 0.05 was considered statistically significant

**FIGURE 8:**
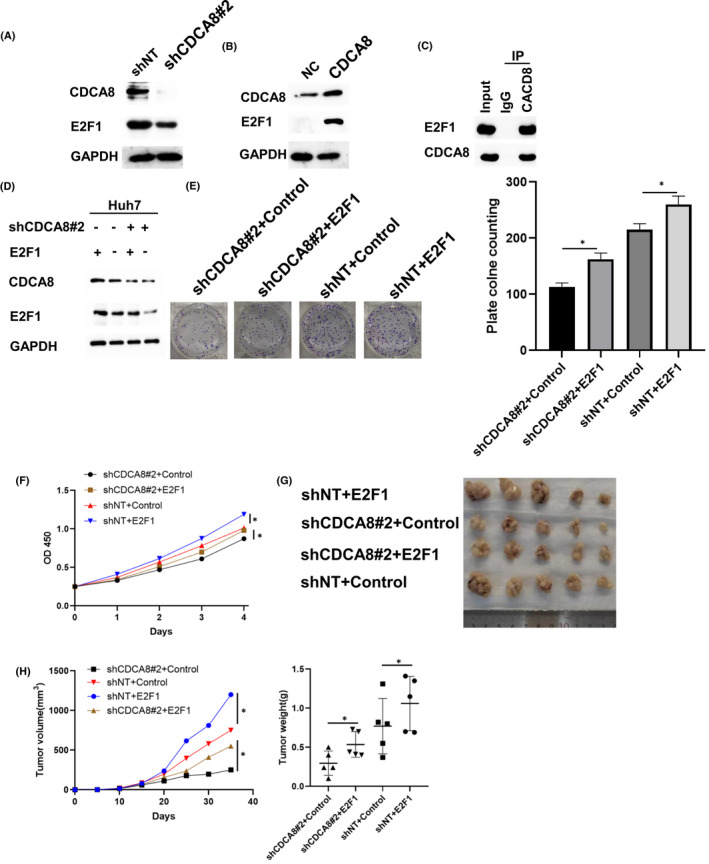
CDCA8 altered the proliferation of HCC cells by regulating E2F1. (A) Upregulation of CDCA8 expression decreased E2F1 expression level. (B) Overexpression of CDCA8 increased E2F1 expression. (C) CDCA8 was found to interact with E2F1. (D) E2F1 expression was overexpressed in shCDCA8 group and shNT group. (E) Results of plate cloning assay showed that recovery of E2F1 expression increased the proliferation of MHCC‐LM3 cells. (F) The CCK‐8 assay indicated that recovery of E2F1 expression increased the proliferation of MHCC‐LM3 cells. (G, H) Recovery of E2F1 expression rescued tumour volume and weight of xenograft in vivo. Images showing the size of tumours in mice. (n = 5) ^*^ *p *< 0.05, ^**^ *p *< 0.01 and ^***^ *p *< 0.001. *p *< 0.05 were considered statistically significant

In 20 pairs of HCC tissues, CDCA8 mRNA expression levels were found to be upregulated (Figure 7A). Moreover, protein levels of CDCA8 were upregulated in 6 HCC pairs (Figure 7B). To verify the significance of CDCA8 in HCC cell proliferation and cell cycle progression, CDCA8 levels in HCC cell lines were evaluated (Figure 7C). First, Huh7 and Hep‐3B cell lines were infected with shCDCA8 and shNT lentivirus (Figure 7D). Through CCK‐8 and colony formation assays, growth and proliferation rates of the shCDCA8 group were significantly lower than those of the shNT group (Figure 7E, F). The apoptosis assays confirmed that there were no differences in apoptotic levels between the shCDCA8 and shNT groups (Supplement Material Figure 2). Then, MHCC‐LM3 wells were infected with CDCA8 and NC lentivirus (Figure 7G). Through the CCK‐8 and colony formation assays, growth and proliferation rates of the CDCA8 group were significantly improved, relative to the NC group (Figure 7H, I). EDU assays were performed to evaluate the effects of CDCA8 knockdown and overexpression on the proliferative capacity of cells (Figure 7J). Moreover, flow cytometry was used to determine whether cell proliferation was influenced by cell cycle distribution. Cells in the shCDCA8 group were arrested at the G0/G1 phase (Figure 7K). Interestingly, the arrest in the G0/G1 phase is P53‐dependent (Supplement Material Figure S3). To confirm the effects of CDCA8 in HCC in vivo, a xenograft model was established using shCDCA8 and shNT stable strains constructed from the Huh7 cell line, respectively (Figure 7L). After 36 days, the nude mice were sacrificed, xenograft tumours were excised and analysed. Tumours from the shCDCA8 group exhibited a significant decrease in weight and volume relative to the shNT group (Figure 7N). Moreover, xenograft models were established using CDCA8 and NC stable strains constructed from the MHCC‐LM3 cell line (Figure 7M) and tumours from the CDCA8 group showed a significant increase in weight and volume relative to the NC group (Figure 7O). Finally, expression levels of cell cycle‐related markers of shCDCA8 and shNT were investigated (Figure 7P). Compared to the shNT group, expression levels of Cyclin A2, Cyclin D1, CDK4, CDK6, Ki67 and PCNA were down‐regulated in the shCDCA8 group (Figure 7P). We found that CDCA8 levels had an effect on E2F1 levels (Figure 8A, B). In the Huh7 cell line, CDCA8 interacted with E2F1 (Figure 8C). We overexpressed E2F1 in the shCDCA8#2 and shNT cell groups, respectively (Figure 8D). Then, we evaluated whether overexpressed E2F1 in shCDCA8#2 and shNT groups affected cell proliferation rates. Overexpressed E2F1 rescued the proliferative ability of shCDCA8#2 group cells (Figure 8E, F). In vivo recovery of E2F1 expression levels also rescued the tumour proliferation inhibition caused by CDCA8 knockdown (Figure 8G, H).

## 4 DISCUSSION

Clinically, in advanced stages, HCC is associated with a rapid proliferation rate and multiple metastatic patterns. Delayed diagnoses lead to poor prognostic outcomes.^15^ Early HCC diagnosis is essential for clinical management of HCC. The current screening methods for HCC, including ultrasonography and AFP, are not sensitive and specific enough to detect early lesions.^16^ Therefore, identification of biomarkers for early‐stage HCC will significantly improve its diagnosis.

In this study, CDCA8 levels were found to be upregulated in HCC tissues. Interestingly, elevated CDCA8 levels were associated with significantly poor OS and PFS outcomes for HCC patients. Furthermore, upregulated CDCA8 levels in HCC were significantly correlated with tumour stage, pathological stage and advanced grade. Expression levels of CDCA8 in HCC and adjacent normal tissues were verified by qPCR, WB and IHC staining assay. Upregulated CDCA8 levels were associated with poor TNM stage and a large HCC tumour volume, consistent with our previous bioinformatics analysis results.

CDCA8 is a member of primary regulators of cell mitosis.^9^ In this study, CDCA8 knockdown inhibited HCC cell proliferation in vivo and in vitro. Through CDCA8 suppression, the cell cycle of Huh7 cells was blocked at the G0/G1 phase. Therefore, CDCA8 regulated HCC cell proliferation by acting on the cell cycle. CDCA8 plays essential roles in various tumour‐related processes. In pancreatic ductal adenocarcinoma, CDCA8 mediates the upregulation of KIF18B and promotes tumour cell proliferation.^17^ Depletion of CDCA8 leads to cell cycle arrest at the G2/M phase, increases DNA damage and apoptosis, and enhances the sensitivity of ovarian cancer cells to Cisplatin and Olaparib.^18^ Through the ROCK signalling pathway, CDCA8 knockdown might inhibit cancer cell proliferation and invasion.^19^ However, the significance of CDCA8 in HCC has not been fully elucidated.

CDCA8 can significantly activate the E2F‐associated pathway in HCC. The E2F family is a group of encoded transcription factor genes involved in the proliferation of eukaryotic cells.^20^ Currently, the E2F family is subdivided into two categories based on members’ functional characteristics: transcriptional activators (E2F1, E2F2 and E2F3a) and transcriptional repressors (E2F3b, E2F4, E2F5, E2F6, E2F7 and E2F8).^21^ Upregulated CDCA8 promotes HCC cell proliferation. Therefore, we evaluated the roles of E2F1, E2F2 and E2F3a in HCC. E2F1 regulated the G1/S phase of the cell cycle by mediating chromosomal DNA replication and gene promoters.^22^ E2F1 is involved in regulation of the proliferation of retinoblastoma in a cell cycle‐dependent manner. In p53‐deficient colon cancer cell lines, Mdm2 inhibition activated E2F1 and enhanced p73‐mediated expressions of Siva‐1 and PUMA to trigger apoptosis.^23^ Previously, E2F1 was found to be significantly upregulated in colon cancer,^24^ however, its levels were not correlated with colon cancer stage and OS, implying that E2F1 may be an important factor in tumorigenesis induction. E2F2 plays a dual role in tumour progression. It can maintain cells in a quiescent state by inhibiting cell cycle regulators and downregulating myc, thereby suppressing tumorigenesis and proliferation.^25^ E2F2 can also act as an ‘activator’ to increase the expression of its target genes.^26^ MiR‐490‐5P prevents HCC metastasis by mediating the downregulation of E2F2 and ECT2,^27^ while LBX2‐AS1 has been shown to enhance E2F2 gene expression to promote ovarian cancer progression.^28^ E2F3 is critical in regulating the transcriptional activation of various oncogenes, which control the rate of tumour and progenitor cell proliferation.^29^ Circ_PGPEP1 promotes gastric cancer proliferation, invasion and migration by regulating E2F3 levels.^30^ MiR‐145‐5P inhibits osteosarcoma cell proliferation by targeting inhibit of E2F3b, which affected Cyclin D1, CDK2, CDK4 and CDK6 expression.^31^ Our results were consistent with those of previous studies.

Under various conditions, the tumour immune microenvironment (TIME) regulates cancer progression. As an inflammation‐associated tumour, the immune‐suppressive microenvironment of HCC contributes to immune tolerance and avoidance.^32^, ^33^ The cell cycle is associated with increased anti‐tumour immunity in multiple cancers.^34^, ^35^, ^36^, ^37^, ^38^ Specifically, CDK4/6 inhibition can induce a diminished tumour immunity in various cancers.^37^ Interestingly, there is a correlation between the E2F protein family and tumour immunity. For example, E2F1 upregulates the expression of Treg cell surface receptors, CD39 and CD73, and further promotes Treg infiltrations.^39^ E2F2‐deficient T lymphocytes exhibit elevated T cell receptor‐induced proliferation, which may contribute to autoimmunity in E2F2‐deficient mice.^40^ In osteoarthritis, E2F3 is positively correlated with multiple immune cells, including resting mast cells, T regulatory cells, CD4 resting memory T cells and activated NK cells.^41^ Therefore, we postulate that CDCA8 mediates cell cycle regulation by affecting E2F functions and HCC immunity. Due to its significant association with the E2F and CDK pathways, it should be determined whether CDCA8 is a potential immune checkpoint. Although the role of CDCA8 involvement in HCC has previously been reported, our results confirm the conclusion of Shuai et al.^42^

## 5 CONCLUSIONS

Compared to adjacent normal tissues, expression levels of CDCA8 were elevated in HCC tissues. Elevated CDCA8 levels were associated with poor OS, PFS and clinical features, including TNM stages, grades and tumour size. Therefore, we postulate that CDCA8 promotes tumour growth through the cell cycle. Besides, CDCA8 has a significant role in HCC‐related immunity. Consequently, CDCA8 may be a potential biomarker for early HCC diagnosis and prognostic prediction.

## DISCLOSURE

The authors declare that they have no competing interests.

## AUTHOR CONTRIBUTION

**Xiaohan Cui:** Formal analysis (equal); Project administration (lead); Writing‐original draft (lead). **Qiu‐Ju Peng:** Data curation (equal); Writing‐review & editing (supporting). **Renzhi Li:** Data curation (equal); Writing‐review & editing (equal). **Xiajie Lyu:** Funding acquisition (equal); Supervision (equal). **Xihu Qin:** Project administration (lead). **Chunfu Zhu:** Funding acquisition (equal); Supervision (equal).

## ETHICAL APPROVAL

This study was approved by the Research Ethics Committee of the Second People's Hospital of Changzhou, China. All participants provided written informed consents.

## Supporting information

Fig S1‐S3

Table S1

## Data Availability

All relevant data from this research are available from the corresponding author upon a reasonable request.
