# Supplementary figures and images for: Cell division cycle associated 8: A novel diagnostic and prognostic biomarker for hepatocellular carcinoma
Source: J Cell Mol Med. 2021 Nov 5;25(24):11097–112. doi: 10.1111/jcmm.17032 (PMC8650035; doi:10.1111/jcmm.17032)

**A**

**Normal group**

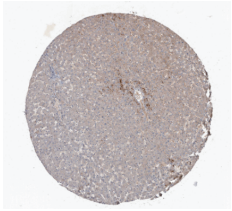

**N1**

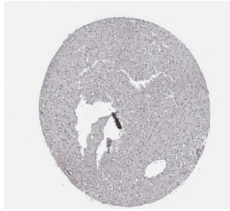

**N2**

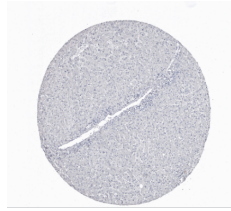

**N3**

**B**

**Tumor group**

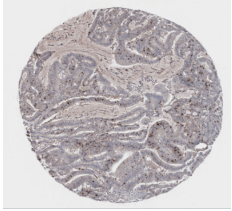

**T1**

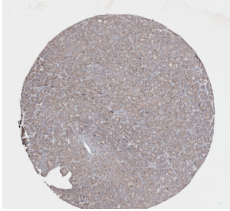

**T2**

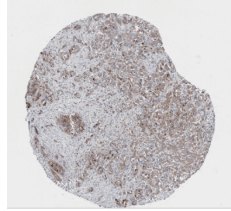

**T3**

**A****shNT**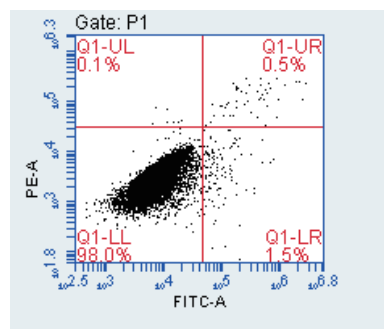**shCDCA8**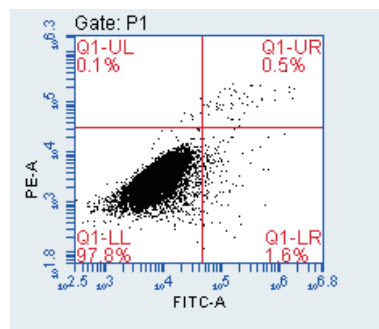

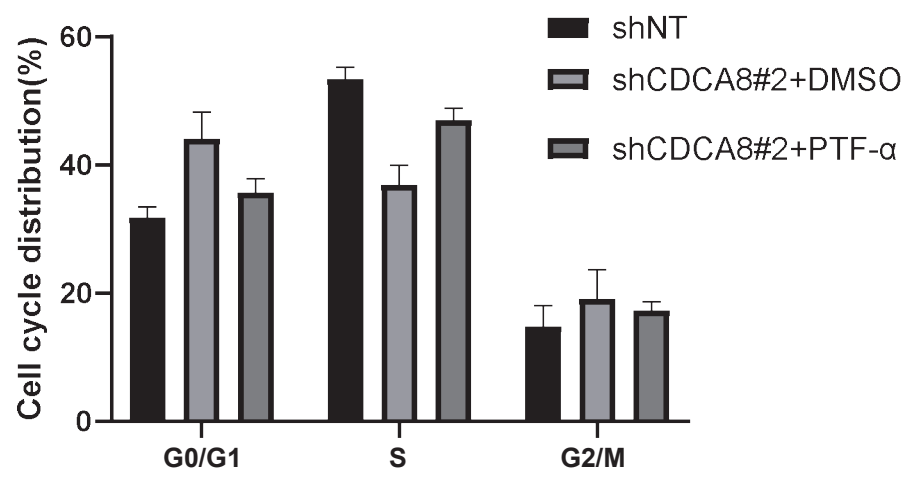

Supplementary Materials Figure 3

Supplement: Supplementary file 1 — Fig S1‐S3 [file JCMM-25-11097-s002.pdf]
