# Supplementary material for: Cell division cycle associated 8: A novel diagnostic and prognostic biomarker for hepatocellular carcinoma
Source: J Cell Mol Med. 2021 Nov 5;25(24):11097–112. doi: 10.1111/jcmm.17032 (PMC8650035; doi:10.1111/jcmm.17032)
Supplement: Supplementary file 2 — Table S1 [file JCMM-25-11097-s001.docx]

| Table S1: Primers and shRNA sequences used in this research (5’-3’) | |
| --- | --- |
| Name | Sequence |
| CDCA8 forward | 5′- CACAGCGAGGTTTTGCTCAG-3′ |
| CDCA8 reverse | 5′- AACTGGGTAGGGACGAGGAG-3′ |
| GAPDH forward | 5′-AAGGTGAAGGTCGGAGTCAAC-3′ |
| GAPDH reverse | 5′-GGGGTCATTGATGGCAACAATA-3′ |
| CDCA8 shRNA | 5′-GCTGTCTACCAAAGTGACGCT-3′ |
